# Supplementary material for: A Critical View of the Application of the APEX Software (Aqueous Photochemistry of Environmentally-Occurring Xenobiotics) to Predict Photoreaction Kinetics in Surface Freshwaters
Source: Molecules. 2019 Dec 18;25(1):9. doi: 10.3390/molecules25010009 (PMC7017383; doi:10.3390/molecules25010009)
Supplement: Supplementary file 1 [file molecules-25-00009-s001.zip › 3.2.4_gcc-4.4.0.zip!/3.2.4_gcc-4.4.0/3.2.4_gcc-4.4.0/doc/octave/html/interpreter/Functions-and-Scripts.html]

Functions and Scripts - Untitled


Next: Errors and Warnings,
Previous: Statements,
Up: Top


---

## 11 Functions and Scripts

Complicated Octave programs can often be simplified by defining
functions. Functions can be defined directly on the command line during
interactive Octave sessions, or in external files, and can be called just
like built-in functions.

- Defining Functions- Multiple Return Values- Variable-length Argument Lists- Variable-length Return Lists- Returning From a Function- Default Arguments- Function Files- Script Files- Function Handles Inline Functions and Anonymous Functions- Commands- Organization of Functions
